# Age-Related Variations of Rabbit Corneal Geometrical and Clinical Biomechanical Parameters

**DOI:** 10.1155/2017/3684971

**Published:** 2017-08-13

**Authors:** Haixia Zhang, Xiao Qin, Xiaomeng Cao, Di Zhang, Lin Li

**Affiliations:** ^1^School of Biomedical Engineering, Capital Medical University, Beijing 100069, China; ^2^Beijing Key Laboratory of Fundamental Research on Biomechanics in Clinical Application, Capital Medical University, Beijing 100069, China

## Abstract

**Purpose:**

To study the variations in corneal clinical biomechanical parameters (CCBP) and corneal geometrical parameters of rabbit in relation to age.

**Methods:**

Rabbits aged 3, 7, 12, 18, and 24 months were enrolled. Each eye of the rabbits was tested with Ocular Response Analyzer (ORA), Optical Coherence Tomography (OCT), and Pachymeter to obtain the intraocular pressure (IOP): Goldmann-correlated IOP (IOPg) and Corneal Compensated Intraocular Pressure (IOPcc); CCBP: Corneal Hysteresis (CH) and Corneal Resistance Factor (CRF); corneal geometric parameters: corneal curvature radius (CCR) and central corneal thickness (CCT).

**Results:**

The IOP of the rabbits changes slightly from 3 to 7 months of age, while it significantly decreases from 7 to 18 months of age and increases from 18 to 24 months of age; CH and CRF decrease with the increase of age; CCT increases from 7 to 18 months and decreases from 3 to 7 months of age and from 18 to 24 months of age; CCR presents an upward trend from 3 to 18 months and a significant decrease between 18 and 24 months of age.

**Conclusion:**

CH and CRF are negatively correlated with age. CCT and CCR are positively correlated with age.

## 1. Introduction

The cornea is a soft tissue located in the outer layer of the eyeball. The transparent cornea provides 70% ocular refractive power [1, 2]. Cornea can not only guarantee the external light to project to the retina directly, but also play an important role in maintaining the normal shape of the eyeball [2]. The maintenance of normal corneal morphology has great significance in the prevention of myopia and keratoconus, and the changes of corneal morphology under different IOPs are closely related to corneal biomechanical properties. Therefore, it is of great importance to study the corneal biomechanical properties for the maintenance of corneal shape, measurement of IOP, and the design of refractive surgery.

**Figure 1:**
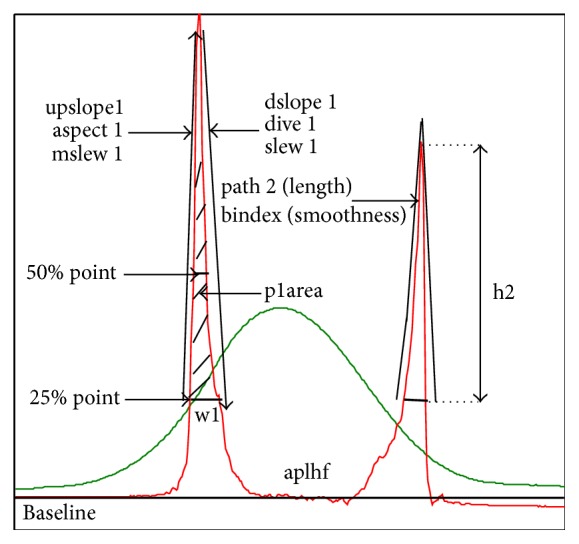
Waveform of ORA examination [15].

As a soft tissue, the heterogeneity of the cornea on the microstructure and morphology results in its nonlinear elastic, anisotropic, and viscoelastic properties. The corneal biomechanical properties can be partially understood by experiments in vitro or experiments in vivo. Although experiments in vitro [3–6] can be carried out easily, it changes the normal physiological shape of the cornea, which will affect the recognition of corneal biomechanical properties and cannot be used in clinical practice directly. Therefore, evaluation of corneal biomechanical properties in vivo attracts more and more attention. Ocular Response Analyzer (ORA, Reichert Inc., Depew, NY) and Corneal Visualization Scheimpflug Technology (Corvis ST) are the most commonly used devices to evaluate corneal biomechanical properties in vivo. Corvis ST is a noncontact tonometer that allows seeing the reaction of the cornea on air impulse to be imaged and provides IOP, corneal Pachymeter, and some biomechanical related parameters such as deformation amplitude (DA) and first applanation time (1st A-time). ORA applies similar principle and reports two corneal biomechanical parameters termed Corneal Hysteresis (CH) and Corneal Resistance Factor (CRF) (Figure 1). These two devices have their own advantages and limitations [7], and in this study, corneal biomechanical parameters in vivo are obtained based on ORA.

In order to distinguish corneal biomechanical parameters in vivo from the commonly used parameters describing the biomechanical properties of cornea, such as elasticity and viscoelasticity, we call CH and CRF as corneal clinical biomechanical parameters (CCBP). CH is intended to quantify the viscoelastic mechanical damping ability of the cornea [8], and CRF is thought to describe its overall rigidity [9]. The ORA also calculates Goldmann-correlated IOP and corneal compensated IOP (IOPg and IOPcc, resp.). IOPg is analogous to standard NCT-IOP measurements, whereas IOPcc is an IOP estimate that uses a mathematical correction to minimize its corneal dependence [8]. Besides, ORA also provides Waveform Score (WS) to evaluate the reliability of the measurements [10, 11] and 37 parameters to describe the characteristic of the waveform which have been found to provide some useful information for the diagnosis of the corneal disease probably such as keratoconus [12, 13]. The interpretations of the waveform parameters [14] are showed in Figure 1 and abbreviations table at the end of the paper.

As one knows some anatomical and histological changes of cornea occur with age. There should also be some changes in corneal geometrical and clinical biomechanical parameters with age. A large number of clinical studies involved the correlation between ORA parameters and age [4, 16–23], and the results showed that with the increase of age, CH and CRF change correspondingly. At the same time, it is found that the central corneal thickness (CCT), corneal curvature radius (CCR), and other geometrical parameters of the cornea also have a series of changes with the change of age [18]. Besides, studies have also indicated that there is a certain correlation between ORA parameters and geometrical parameters [6, 24–27]. However, there have not been clear biomechanical interpretations of CH and CRF available yet; that is, the relationship between CCBP and corneal biomechanical properties, such as elasticity and viscoelasticity, has not been clear.

If both the ORA parameters-age relations and the corneal biomechanical properties-age relations had been understood comprehensively, it is possible to study the biomechanical interpretations of CH and CRF by combining these two relationships. Besides, comparison of the ORA parameters-age relations and the corneal biomechanical properties-age relations will contribute to understanding the characteristic of CH and CRF in keratoconus patients and patients after refractive surgery, because the development of the keratoconus and the individualized design of corneal refractive surgery are closely related to corneal biomechanical properties [28, 29]. Following this idea, we need to pay more attention to the biomechanical properties-age relations since there have been a number of results on ORA parameters-age relations and the age of the subjects ranged from 4 years to 91 years [4, 16–23]. However, human cornea is too precious to study corneal biomechanical properties-age relations using experimental test in vitro. Elsheikh et al. had studied the human corneal biomechanical properties aged between 50 and 95 years [3], but few researches are related to the younger's, which made it difficult to understand corneal biomechanical properties from their CCBP. This makes us pay more attention to teenagers because some corneal diseases such as myopia and keratoconus are observed in adolescence.

As we know, rabbit eyes are similar to human eyes in size [30, 31], offering advantages in the evaluation of new drugs and surgical procedures. If we further assume that corneal development process of human and rabbit is consistent, it is reasonable to study the relationship between rabbit corneal biomechanical properties and age, and the relationship between rabbit CCBP and age. There have been results of biomechanical properties-age relations [4, 5], but little ORA examination results of rabbits were reported in literature. Therefore, this study focuses on the age-related changes of CCBP and corneal geometrical parameters by applying ORA, Optical Coherence Tomography MARK II (OCT, TOPCON, Japan), and Pachymeter (Reichert Inc., Depew, NY) to rabbits corneas with different ages. The significance of this study lies in providing normal CCBP of rabbits with different ages and may provide useful information in exploring the biomechanical interpretations of CH and CRF.

## 2. Methods

### 2.1. Subjects and Measurements

30 New Zealand white rabbits aged 3, 7, 12, 18, and 24 months (6 for each age) with healthy eyes were enrolled in the study. All of the rabbits were provided by Animal Laboratory Center of Capital Medical University, and the experiments were followed by the “Regulation of Laboratory Animal Management.” Every rabbit was examined as follows.

*Preparation before Experiment*. Weigh the rabbits and inject 3% pentobarbital sodium (Merck, Germany) at a dose of 1 ml/kg into the rabbit ear edge vein of the rabbits to achieve the general anesthesia. 

*ORA Examination*. When the rabbit was fixed in front of the machine with its eye fixating on the green light, trigger the air pulse and carry out continuous 4 measures, and then two IOP values (IOPg and IOPcc) and CCBP were recorded. 

*OCT Examination*. Fix the eye in front of the camera and make sure the vertex of the cornea is at the center of the scanning. Select anterior module of the OCT and press the scan button to get a set of clear corneal morphology images, which will be used to calculate the 4 typical corneal curvature radii (curvature radius on the horizontal and vertical direction of the anterior surface and posterior surface of the cornea) and corneal central thickness. 

*Pachymeter Measurement*. Place the rabbit on the experimental platform to measure the corneal central thickness by Pachymeter. During the measurement, the ultrasonic probe should contact with the corneal vertex vertically and the measurement was repeated for 3 times continuously. The results were used as gold standard to verify the reliability of corneal geometrical parameters extracted by OCT test results.

### 2.2. Calculation of Geometrical Parameters

**Figure 2:**
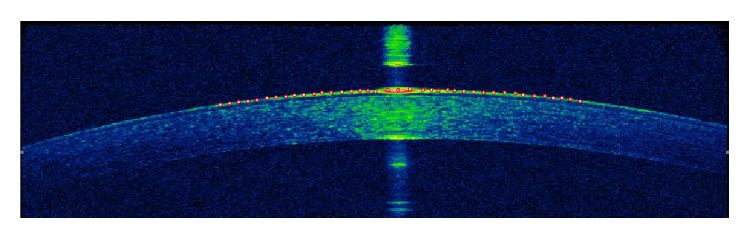
Calculate geometrical parameters from corneal morphology image.

The method we took to calculate the CCR from corneal morphology image captured by the OCT is in the following (Figure 2). Firstly, read the picture into the Mathematica™ (Wolfram Research, Inc., Champaign, IL, USA) and extract the coordinates of the corneal boundary at the center of the cornea (diameter 3 mm) with the coordinate tools. Then the quadric curve (1) was used to fit the data, and finally calculate the corneal curvature according to the curvature formula (2).

(1) fx,y=c20x2+2c11xy+c02y2+2c10x+2c01y+c00,

(2) c=c0c01+c11x+c02y3+4c02c11c10+c20x+c11y3c01+c11x+c02yc01+c11x+c02y2+c10+c20x+c11y23/2.

In the same way, the corneal central thickness can be calculated by extracting the coordinates of the vertices of the inner and outer surfaces of the cornea.

### 2.3. Statistical Analysis

All of the 30 rabbits' experimental results, including intraocular pressure (IOP), CCBP, CCR, CCT, and other ORA waveform parameters, were executed by Kolmogorov-Smirnov (K-S) normality test separately. For parameters subjected normal distribution, one-way ANOVA test was used to determine whether there is statistical difference among different groups, and Least Significant Difference (LSD) test was used for further analysis of the parameters between two groups. Further Spearman test was used to analyze the correlation between parameters and age. Besides, correlation analysis was conducted between mechanical parameters and geometrical parameters. All of the statistical analysis was performed using SPSS software (SPSS version 21.0, International Business Machines Corporation, New York, United States of America), and we consider that all of the tests are significant if *p* < 0.05.

## 3. Results

### 3.1. Correlation between ORA Parameters and Age

334 ORA records in total for all of the 30 rabbits were adopted at the screening condition of “*w*s > 3.5” [10, 11]. The mean values of parameters for each eye were involved in the statistical analysis.  Table 1 gives the results of the IOP and CCBP of the rabbit cornea with different ages.

K-S tests for IOPg, IOPcc, CH, and CRF showed that all of the 4 parameters were subjected to normal distribution (*p* > 0.05). The results of the one-way ANOVA test of the 4 parameters showed that there were statistically significant differences among different groups for IOP, CH, and CRF (*p* < 0.05).

Table 2 gives the results of the Spearman test between IOP, CCBP, and age. It indicates that both CH and CRF were negatively correlated with age, while it does not suggest there was significant correlation between IOP and age (*p* > 0.05).

**Figure 3:**
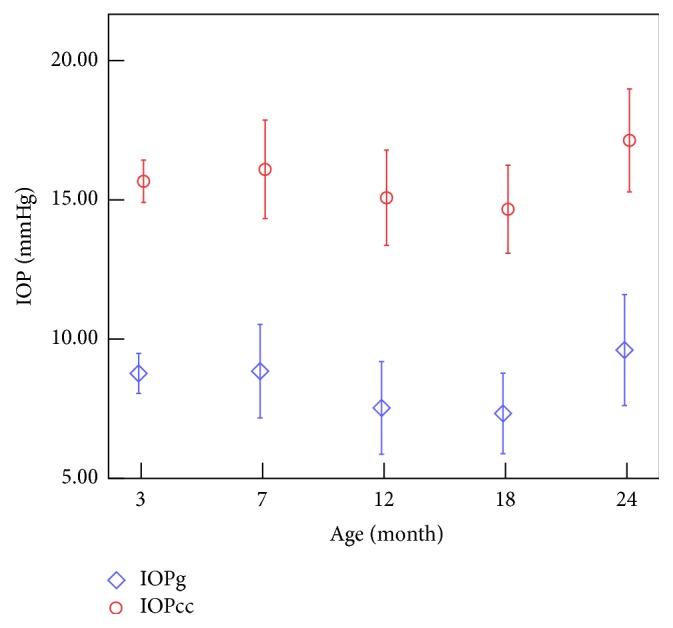
Variations of the IOP with the increase of the age.

**Figure 4:**
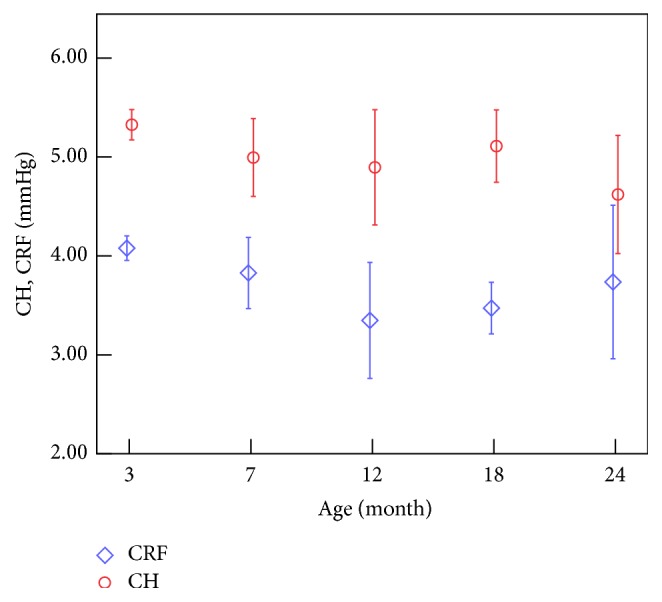
Variations of CH and CRF with the increase of age.

LSD test results of IOP and CCBP are shown in Table 3. And the variations of all the 4 parameters with age were shown in Figures 3 and 4.

From Tables 2 and 3 and Figure 3 we can get that the IOP decreases significantly when the rabbits grow from 7 to 18 months of age and increases from 18 to 24 months of age. And Spearman test showed that IOPg and IOPcc were negatively correlated with age from 7 to 18 months (*r* = −0.431, −0.365; *p* < 0.05). Tables 2 and 3 and Figure 4 tell us that both CH and CRF decrease with the increase of age, and for further comparison we can find that CRF decreases rapidly from 3 months to 12 months of age and the variation after that was not statically significant, while CH decreases significantly from 3 to 7 months of age and 18 to 24 months of age.

**Figure 5:**
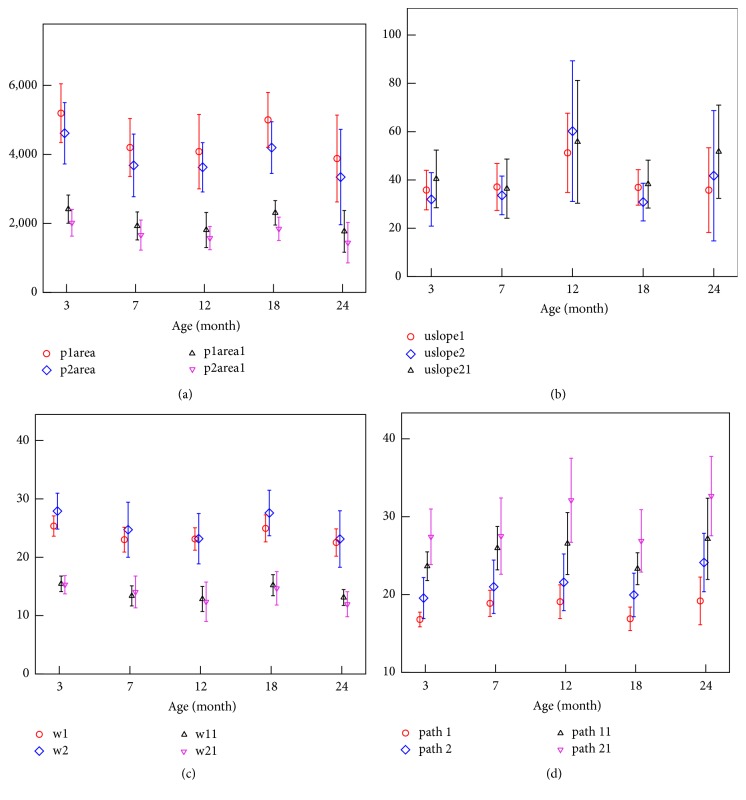
Variations of ORA waveform parameters with the increase of age: the variation of the areas under the peaks (a), the upslope of the peaks (b), the width of the peaks (c), and path length of the peaks (d).

Besides IOP and CCBP, 15 ORA waveform parameters were found to be statistically different among different groups (*p* < 0.05), where it gave in detail p1area (*p* = 0.005), p2area (*p* = 0.018), p1area1 (*p* = 0.002), p2area2 (*p* = 0.018), uslope1 (*p* = 0.028), uslope2 (*p* = 0.003), uslope21 (*p* = 0.023), w1 (*p* = 0.004), w2 (*p* = 0.013), w11 (*p* = 0.000), w21 (*p* = 0.010), path1 (*p* = 0.004), path2 (*p* = 0.011), path11 (*p* = 0.024), and path21 (*p* = 0.004). The variations of all the 15 parameters with age were shown in Figure 5. The results showed that the variation of CH was similar to those of the variations of areas under the peaks in the applanation curve. The variation of CRF was similar to the width of the peaks, while it was opposite to the upslope of the peaks and the path length of the peaks.

### 3.2. Correlation between Geometrical Parameters and Age

**Figure 6:**
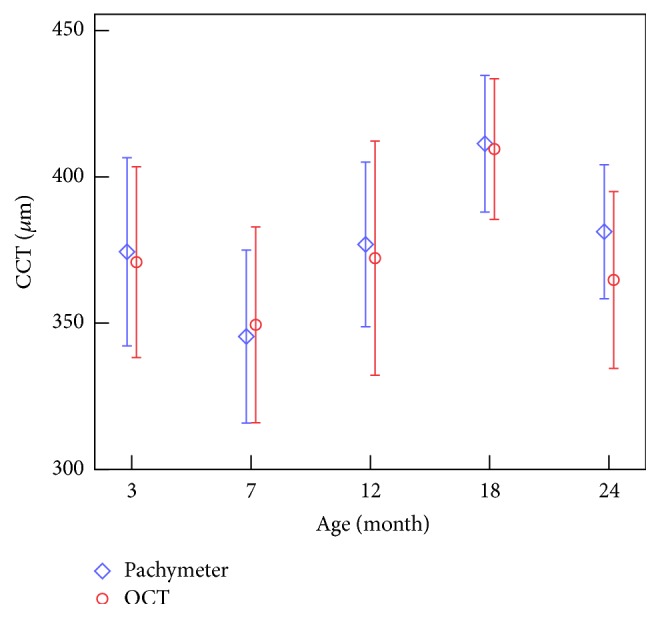
Variation of CCT with the increase of age.

Figure 6 gives the CCT values gained from Pachymeter and calculated by OCT results. There were no significant differences between the two sets (*p* = 0.905), which indicated the reliability of our method to extract the edge of cornea.

The results of the geometrical parameters of the rabbit cornea with different ages are shown in Table 4, where CCR_H1 and CCR_V1 represent the curvature radius on the horizontal and vertical directions of the posterior surface of the cornea, respectively, and CCR_H2 and CCR_V2 represent the curvature radius on the horizontal and vertical directions of the anterior surface of the cornea. Statistical analysis results showed that CCR on the horizontal direction is statistically larger than those on vertical and the CCR of the posterior surface of the cornea is larger than those of the anterior surface (*p* < 0.05).

The K-S Test of the geometrical parameters and the one-way ANOVA test between geometrical parameters and age showed that there are statistically significant differences for CCT and CCR among different groups.  Table 5 gives the results of the correlation between geometrical parameters and age. It showed that both CCT and CCR are positively correlated to age.

**Figure 7:**
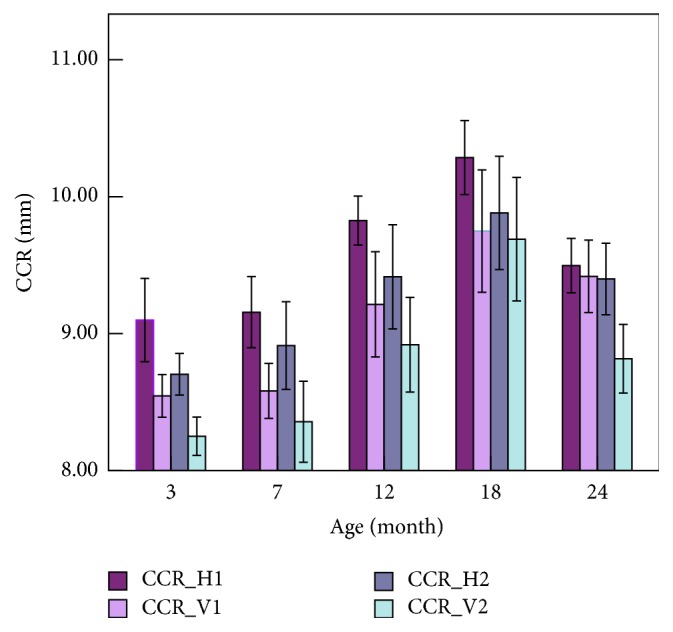
Variations of CCR with the increase of age.

Figures 6 and 7 show the variations of CCT and CCR with age. We can see that CCT increased significantly when the rabbits grow from 7 to 18 months of age while decreased from 3 to 7 and from 18 to 24 months of age, and Spearman test between CCT and age showed similar results (*r* = −0.667; *p* < 0.001) when the rabbits grows from 7 to 18 months. Figure 6 showed an upward trend of the CCR at the age of 3–18 months and a significant decrease between 18 and 24 months of age.

### 3.3. Correlation between ORA Parameters and Geometrical Parameters

The results of the correlation analysis between ORA parameters and geometrical parameters are shown in Table 6 and we can find that CRF was correlated negatively with CCR while significant correlation was not found among other parameters.

## 4. Discussion

In this study, ORA, OCT, and Pachymeter were used to obtain IOP, clinical biomechanical parameters, and geometrical parameters of the cornea to analyze their changes with age. The results showed that both clinical biomechanical parameters and geometrical parameters of rabbit cornea change with increase of age. One of the main innovations is the proposed approach to study the relationship between CCBP and corneal traditional biomechanical parameters by comparing the CCBP-age relations and corneal biomechanical parameters-age relations, which may result in a better understanding of the biomechanical interpretation of CCBP. Another innovation is the given reference values of ORA parameters and geometrical parameters of normal rabbits with different ages. Considering rabbit cornea is one of the most commonly used corneal specimens in researches, the present methods and results are very useful and important to the studies on rabbit cornea, such as the studies of biomechanical properties of rabbit cornea after laser in situ keratomileusis with different repair time [32] and studies on the biomechanical responses to corneal cross-linking in rabbits [33].

According to the relationship between the age of rabbits and human [34], rabbits aged 3, 7, 12, 18, and 24 months roughly corresponds to 5, 11, 18, 25, and 35 years of human, respectively. And in the following discussion, our results on the rabbit corneal biomechanics-age relations are compared with results of the human cornea in previous studies based on this age correspondences.

### 4.1. Correlation among Corneal Geometrical Parameters, IOP, and Age

Table 5 shows that both CCR and CCT are positively related to age. The variations of both the CCR in our study and the volume of the eyeball in [6] with the increases of age are consistent. The CCT of 302 healthy individuals aged 10–69 years [18] showed an upward trend after age 20, which showed a similar trend with our results of rabbits at the age of 12 to 18 months.

The CCR of the anterior surface is larger than that of the posterior surface in human, which is opposite to the rabbit cornea [35]. Considering that the thickness distributions from the center to the limbus of the cornea in human and in rabbit are also opposite to each other, we may surmise that the difference is correlated to their respective corneal physiological characteristics. The CCR on the horizontal direction is larger than that on the vertical direction, which is coincident with human cornea [24].

Although significant linear correlation is not found between IOP and age (Table 2), it can be found that both IOPg and IOPcc decrease when the rabbits grow from 7 to 18 months of age and increase from 18 to 24 months of age. The IOP of rabbit eyeball obtained by anterior chamber perfusion with different ages [6] decreases with the increase of age (3–18 months). Our results of IOPg and IOPcc showed a similar trend with those of them.

The results of the ocular parameters of the young and the elderly subjects [16] showed that the IOPs of the healthy elderly subjects are significantly higher than those of the healthy young subjects, and from Figure 3 and Table 3 we can also get that IOP of the rabbits showed an upward trend when the age increases from 12 months to 24 months. A similar conclusion was found [20] by analyzing the IOPs among the age groups of “<46 years,” “46–55 years,” and “56–65 years.” Research of the age-related changes of the corneal biomechanical parameters and IOP in the Turkish population [17] found a weak negative correlation between IOPg and age while age was found to have no significant effects on IOPcc; the results are coincident with the rabbits' results aged 7–18 months.

Table 6 suggests that the correlation between IOP and CCT was not significant statistically, while from the point of view of age stages, we can observe that IOPg and CCT vary oppositely with the increase of age (Figures 3 and 6), which may remind us that IOPg obtained from ORA may be influenced by CCT.

### 4.2. Correlation between CCBP, ORA Waveform Parameters, and Age

Tables 2 and 3 and Figure 4 show that CH and CRF decrease with the increase of age. Studies have found that CH is negatively correlated with age: *r* = −0.353 for 10–69 years, *r* = −0.17 for 19–89 years, and *r* = −0.372 for 18–59 years [17–19]. The results showed a similar variation of CH to our studies overall. The negative correlation between age and CRF was found both from our study on rabbits and from [17–19] on human cornea. Another study (Strobbe et al., 2014) [20] on 400 human corneas (400 eyes) aged 21–88 years showed that CH had a highest value in young adults (21–46 years) and a lowest value in the oldest subjects (>75 years), while CRF had no significant difference among different age groups. The similar results are found in our results; that is, after a decrease from 3 to 12 months of age CRF shows a relative stable trend of variations, and CH values have two decrease stages, which means that there is a maxima and a minimum at least during the development.

Focusing on rabbit cornea falling 3–18 months, CH decreases significantly from 3 to 7 months of age; results of stress relaxation of rabbit corneal strips have also showed a significant variation from 3 to 7 months of age [5]. These 2 facts suggested a significant variation in rabbit corneal viscoelasticity from 3 to 7 months because of the corneal development. CH was relatively stable from 7 to 18 months; this result is not different from Kirwan's result on the children aged 4–18 years old [36]. Another significant decrease in CH from 18 to 24 months needs to be further investigated.

CH, one of the CCBP gained from ORA, is related to the Corneal Hysteresis and may reflect the viscoelastic properties of the cornea [8]. CRF, another of CCBP, represents the corneal resistance factor and may reflect the overall stiffness of the cornea [9]. Following the negative correlation between CRF and age, we can infer that the corneal stiffness varies with the increase of age. Corneal uniaxial tensile test [5] of the rabbits aged 3 and 7 months of age showed that the tangent modulus increased slightly with the increase of age. From the negative correlation between CH and age, it implies that cornea exhibits different viscoelastic properties. The stress relaxation of corneal strips [5] shows that the stress relaxation in rabbits aged 7 months was significant faster than those of the 3-month-old rabbits. If the difference of tests between in vivo and in vitro was neglected, these results may indicate that lager CRF and CH maybe correspond to lower corneal stiffness and slower stress relaxation, respectively. The deduction is consistent with the following researches: CRF value is lower after LASIK [37] and there is a significant increase in rabbit corneal elastic modulus after LASIK [32]. However, more experimental data from the tests on both in vitro and in vivo are expected to obtain more precise relationship.

Ref. [7] reports that a reduced applanation (optical reflectance) signal width and/or amplitude perhaps indicate increased localized deformation, which would cause the optical detector to see a smaller reflectance area, similar to the corneal surface after LASIK [7]. ORA waveform of keratoconus patient showed a similar characteristic [11, 12]. According to Figure 5, the areas under the peaks of the ORA applanation curve have a significant decrease from 3 to 7 months of age; this may be related to the smoothness of anterior surface of the cornea and the nonuniformity of the corneal thickness. Keeping the opposite variation between the area under the peaks and CCT (Figure 6) in mind, we speculate that this decrease is possibly caused by the shorter time that the cornea keeps the flat state in for 7-month-old rabbit than 3-month-old rabbit because of a thinner cornea. According to the results that the variation of CRF was similar to the width of the peaks in the applanation curve, while it was opposite to the upslope of the peaks and the path length of the peak, we guess the width of the peaks in the applanation curve, the upslope of the peaks, and the path length of the peak may be related to the stiffness of the cornea because these parameters reflected the ability of the cornea to deform under external force.

### 4.3. Correlation between ORA Parameters and Geometrical Parameters

When comparing the results of the correlation between CCBP and geometrical parameters we can find that CRF was correlated negatively with CCR, while significant correlation was not found between CH and CCR, CH and CCT, and CRF and CCT. Studies on clinical ORA data of the human have not reached to an agreement (CH and CRF were negatively correlated with CCR [17, 27], and no significant correlation between CH, CRF, and CCR [18, 29, 30]). Our research of the rabbit cornea has got similar results with [25, 26]; that is CRF was correlated negatively with CCR, while CH was not correlated with CCR. There is a positive correlation between CCBP and CCT of the human cornea [24, 38–40] while the correlation is not found in rabbit. The main reasons may be the differences of the corneal geometrical features and corneal pathological status of two kinds of subjects. The human cornea is the thinnest in the center and thicker at the edge, while the rabbits is the opposite, and the different thickness distribution may result in the different correlation between biomechanical parameters and CCT. Moreover, the variations of CCT and CCR in most researches are results of pathology of the eyeball such as myopia and keratoconus, which will change the biomechanics of the cornea accordingly, whereas rabbits involved in our study are healthy.

### 4.4. Implication of the Results to Ophthalmology Clinic

Elevated IOP has been known as the principal risk factor of glaucomatous [41]. In this study, the IOP increases significantly at the age of 24 months, which may explain why humans aged more than 40 years are prone to glaucoma [42, 43]. This study shows that IOPg and CCT vary oppositely with the increase of age; this indicates that the IOP values measured by ORA may be influenced by CCT. The results also remind us to pay attention to the influences of CCT when evaluating IOP in clinical practice.

A large number of clinical studies have found that CH and CRF are lower significantly in patients with myopia [28] and keratoconus [29] than normal cornea. Our results have showed a significant decrease at the stage of about 7 months of age, which indicates that corneal biomechanical properties were in the process of varying obviously at this stage. Combining these two facts, we can infer that using the eyes scientifically and properly is an effective way to prevent myopia for teenagers. Results of our study indicate that it is possible to explain why teenagers are prone to myopia from the aspect of biomechanics.

From Figure 5, the minimum of the peaks' width and the maximum of the peaks' upslope and path length are attained at 12 months. Since these parameters may be related to the stiffness of the cornea, we think that human corneal development may be not stopped until 18 years of age according to the relationship between the age of rabbits and human. So corneal refractive surgery should better be operated at a particular age when the corneal biomechanical status is stable relatively.

### 4.5. Limitations of the Research

There are two limitations of the study. (1) Restricted by lacking of anterior OCT in our laboratory, the CCR obtained by OCT maybe larger than the CCR obtained by keratometer because the scanning area of the OCT is limited. Our research is more interested in the variation of CCR with the increase of age, rather than the absolute value of CCR. Besides the CCT gained from Pachymeter and calculated by OCT results showed no significant difference, which certified the reliability of our method to extract the edge and the fact that the CCR calculated by OCT will be closely correlated to the real CCR. The method will be more practical if the scanning is enlarged. (2) The sample size of our study is smaller compared with clinical studies on the biomechanical characteristics of the human cornea. However, our research has showed a similar results with other studies which reflects that our results with limited samples are credible. Besides, the statistical power results (statistical power > 0.8) of our study showed that our results are statically reliable.

## 5. Conclusion

ORA, OCT, and Pachymeter were used to obtain the clinical biomechanical parameters and geometrical parameters of the cornea in this study, and we can conclude that both CH and CRF are negatively correlated with age and that both CCT and CCR are positively correlated with age. There is no significant correlation between clinical biomechanical parameters (CH, CRF) and geometrical parameters of rabbit cornea.

## Figures and Tables

**Table 1:** IOP and CCBP of rabbit cornea with different ages.

Age (months)	IOPg (mmHg)	IOPcc (mmHg)	CRF (mmHg)	CH (mmHg)
3	8.78 ± 0.71	15.68 ± 0.76	4.08 ± 0.12	5.32 ± 0.15
7	9.41 ± 2.45	16.69 ± 2.85	3.91 ± 0.42	4.89 ± 0.79
12	6.86 ± 1.55	14.56 ± 1.99	3.12 ± 0.28	4.86 ± 0.67
18	7.05 ± 1.75	14.41 ± 1.82	3.41 ± 0.43	5.13 ± 0.44
24	9.57 ± 1.38	17.21 ± 1.82	3.64 ± 0.46	4.53 ± 0.74

**Table 2:** The results of the correlation between IOP, CCBP, and age.

Parameters	IOPg	IOPcc	CRF	CH
*r*	−0.176	0.087	−0.479	−0.179
*p*	0.086	0.400	0.000^*∗*^	0.032^*∗*^

^*∗*^The correlation is statistically significant.

**Table 3:** Least Significant Difference (LSD) test results of IOP and CCBP.

Age (month)	IOPg (mmHg)		IOPcc (mmHg)		CRF (mmHg)		CH (mmHg)	
	Mean difference	*p*	Mean difference	*p*	Mean difference	*p*	Mean difference	*p*
3–7	−0.638 ± 0.642	0.323	−1.011 ± 0.745	0.178	0.171 ± 0.128	0.184	0.430 ± 0.214	0.048^*∗*^
3–12	1.916 ± 0.835	0.024^*∗*^	1.118 ± 0.969	0.251	0.957 ± 0.166	0.000^*∗*^	0.462 ± 0.279	0.102
3–18	1.721 ± 0.700	0.016^*∗*^	1.266 ± 0.812	0.123	0.672 ± 0.139	0.000^*∗*^	0.194 ± 0.234	0.408
3–24	−0.791 ± 0.796	0.323	−1.530 ± 0.924	0.101	0.435 ± 0.159	0.007^*∗*^	0.797 ± 0.266	0.004
7–12	2.553 ± 0.690	0.000^*∗*^	2.130 ± 0.800	0.009^*∗*^	0.786 ± 0.137	0.000^*∗*^	0.032 ± 0.230	0.891
7–18	2.359 ± 0.518	0.000^*∗*^	2.277 ± 0.601	0.000^*∗*^	0.501 ± 0.103	0.000^*∗*^	−0.235 ± 0.173	0.177
7–24	−0.153 ± 0.642	0.812	−0.518 ± 0.745	0.488	0.264 ± 0.128	0.042^*∗*^	0.367 ± 0.214	0.091
12–18	0.194 ± 0.744	0.795	0.147 ± 0.863	0.865	−0.285 ± 0.148	0.057	−0.267 ± 0.249	0.285
12–24	−2.707 ± 0.835	0.002^*∗*^	−2.648 ± 0.969	0.008^*∗*^	−0.522 ± 0.166	0.002^*∗*^	0.335 ± 0.279	0.233
18–24	−2.512 ± 0.700	0.001^*∗*^	−2.795 ± 0.812	0.001^*∗*^	−0.236 ± 0.139	0.093	0.602 ± 0.234	0.012^*∗*^

^*∗*^The difference is statistically significant.

**Table 4:** Geometrical parameters of rabbit cornea with different ages.

Age (month)	CCT (*μ*m)	CCR_H1 (mm)	CCR_H2 (mm)	CCR_V1 (mm)	CCR_V2 (mm)
3	374 ± 32	9.10 ± 0.48	8.70 ± 0.24	8.55 ± 0.25	8.25 ± 0.22
7	362 ± 27	9.45 ± 0.46	9.20 ± 0.48	8.80 ± 0.46	8.47 ± 0.40
12	377 ± 28	9.83 ± 0.25	9.42 ± 0.53	9.21 ± 0.54	8.92 ± 0.48
18	411 ± 19	10.31 ± 0.38	9.93 ± 0.61	9.84 ± 0.62	9.76 ± 0.77
24	381 ± 23	9.50 ± 0.31	9.40 ± 0.41	9.42 ± 0.42	8.82 ± 0.39

**Table 5:** The results of correlation between CCR, CCT, and age.

Parameters	CCR_H1	CCR_H2	CCR_V1	CCR_V2	CCT
*r*	0.475	0.490	0.643	0.597	397
*p*	0.000^*∗*^	0.000^*∗*^	0.000^*∗*^	0.000^*∗*^	0.000^*∗*^

^*∗*^The correlation is statistically significant.

**Table 6:** The results of the correlation between biomechanical parameters and geometrical parameters.

	IOPg		IOPcc		CRF		CH	
	*r*	*p*	*r*	*p*	*r*	*p*	*r*	*p*
CCR_H1	−0.135	0.188	−0.059	0.567	−0.359	0.000^*∗*^	−0.149	0.148
CCR_H2	−0.094	0.361	0.033	0.748	−0.282	0.005^*∗*^	−0.128	0.214
CCR_V1	−0.083	0.422	0.016	0.875	−0.294	0.004^*∗*^	−0.158	0.125
CCR_V2	−0.198	0.053	0.126	0.220	−0.370	0.000^*∗*^	−0.082	0.427
CCT	−0.158	0.125	−0.126	0.220	−0.194	0.058	0.017	0.866

^*∗*^The correlation is statistically significant.

## Abbreviations

ORA: Ocular Response Analyzer
CCBP: Corneal clinical biomechanical parameters
OCT: Optical Coherence Tomography
IOP: Intraocular pressure
IOPg: Goldmann-correlated IOP
IOPcc: Corneal compensated IOP
CH: Corneal Hysteresis
CRF: Corneal Resistance Factor
CCT: Corneal Central Thickness
CCR: Corneal Curvature Radius
CCR_H1: Horizontal CCR of the posterior surface of the cornea
CCR_H2: Horizontal CCR of the anterior surface of the cornea
CCR_V1: Vertical CCR of the posterior surface of the cornea
CCR_V2: Vertical CCR of the anterior surface of the cornea
p1area, p2area: Upper 75% area of the applanation peak1 and peak2, respectively
p1area1, p2area1: Upper 50% area of the applanation peak1 and peak2, respectively
w1, w2: Width of peak1 and peak2 at point of 50% of the base region
w11, w21: Width of peak1 and peak2 at point of 25% of the base region
uslope1, uslope2: Rate of increase from 25% point to the peak1 and peak2
uslope11, uslope21: Rate of increase from 50% point to the peak1 and peak2
path1, path2: Absolute value of path length around peak1 and peak2
path11, path21: Upper 50% of absolute value of path length around peak1 and peak2.
